# Computational Methods for Characterizing Cancer Mutational Heterogeneity

**DOI:** 10.3389/fgene.2017.00083

**Published:** 2017-06-14

**Authors:** Fabio Vandin

**Affiliations:** Department of Information Engineering, University of PadovaPadova, Italy

**Keywords:** cancer heterogeneity, mutations, cancer pathways, mutual exclusivity, clinical association

## Abstract

Advances in DNA sequencing technologies have allowed the characterization of somatic mutations in a large number of cancer genomes at an unprecedented level of detail, revealing the extreme genetic heterogeneity of cancer at two different levels: inter-tumor, with different patients of the same cancer type presenting different collections of somatic mutations, and intra-tumor, with different clones coexisting within the same tumor. Both inter-tumor and intra-tumor heterogeneity have crucial implications for clinical practices. Here, we review computational methods that use somatic alterations measured through next-generation DNA sequencing technologies for characterizing tumor heterogeneity and its association with clinical variables. We first review computational methods for studying inter-tumor heterogeneity, focusing on methods that attempt to summarize cancer heterogeneity by discovering pathways that are commonly mutated across different patients of the same cancer type. We then review computational methods for characterizing intra-tumor heterogeneity using information from bulk sequencing data or from single cell sequencing data. Finally, we present some of the recent computational methodologies that have been proposed to identify and assess the association between inter- or intra-tumor heterogeneity with clinical variables.

## 1. Introduction

Somatic mutations, alterations of the DNA which accumulate during the lifetime of an individual, are the most common cause of cancer. High-throughput sequencing technologies now allow to identify and catalog the entire complement of somatic mutations in a tumor (Mardis, 2008; Meyerson et al., 2010) and many studies, including the ones from TCGA^1^ and ICGC ^2^, have used these technologies to measure mutations in the whole exome or whole genome of hundreds or thousands of tumors (e.g., see The Cancer Genome Atlas Research Network, 2017a,b for recent studies). These studies provide a detailed characterization of the landscape of somatic mutations in cancer, describing the hundreds-thousands of somatic mutations appearing in each tumor. Such somatic mutations include *single nucleotide variants* (SNVs) as well as *copy number aberrations* (CNAs), larger scale events which modify (by amplifications or deletions) the number of copies of a DNA region. Only a handful of all somatic mutations, called *driver* mutations, confer selecting advantage to cancer cells, while most somatic mutations are *passenger* mutations not contributing to the disease (Garraway and Lander, 2013; Vogelstein et al., 2013).

**Figure 1:**
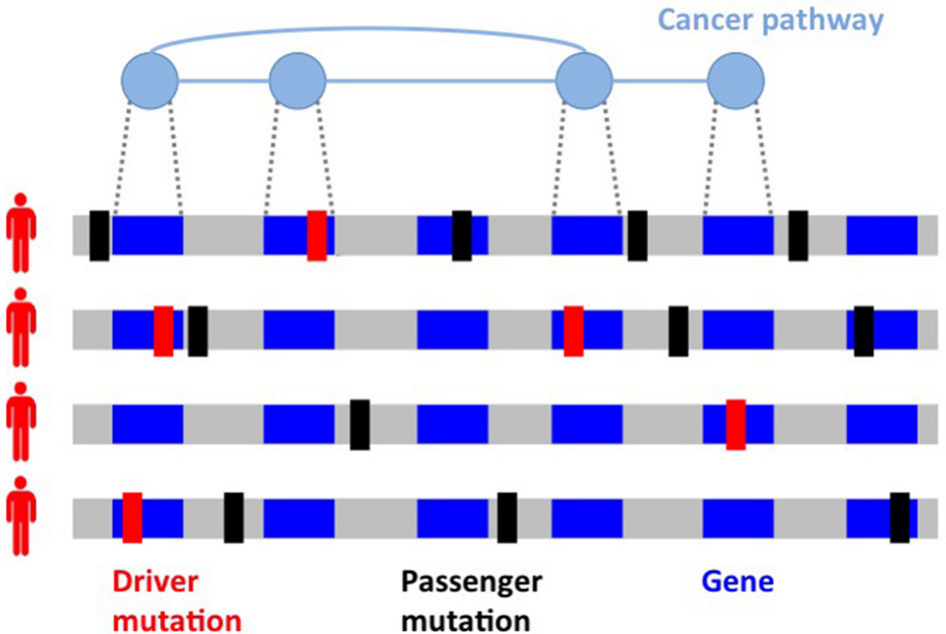
Inter-tumor heterogeneity and its causes. Driver mutations (in red) target genes which are members of different cancer *pathways*, sets of interacting genes which perform specific functions and are altered in cancer. Passenger mutations (in black) not related to the disease comprise the majority of mutations in a tumor. Different mutated genes in cancer pathways and different passenger mutations are observed in tumors of the same type, with two cancer genomes often having no mutation in common.

One of the most striking features of cancer mutational landscape is its *inter-tumor heterogeneity* (Figure 1): no two cancer genomes bear the same collection of somatic mutations, with many pairs of tumors having no mutation in common (Stratton et al., 2009), and a limited number of mutations appear in a large fraction of tumors, with most genes being mutated (by SNVs or CNAs) in <5% of all patients with a given cancer type (Ciriello et al., 2013; Kandoth et al., 2013; Tamborero et al., 2013). Inter-tumor heterogeneity hinders efforts to identify *driver genes*, bearing driver mutations, by detecting frequently mutated genes, i.e., genes mutated in a significantly high fraction of patients (Dees et al., 2012; Lawrence et al., 2013). In addition, frequency-based methods may result in several false positives (D'Antonio and Ciccarelli, 2013) since genomic features not related to the disease, including (normal) gene expression levels and replication time (Lawrence et al., 2013), can nonetheless lead to a high mutation frequency for a gene and must therefore be taken into account to identify significantly mutated genes (Lawrence et al., 2014).

One of the causes of inter-tumor heterogeneity is the fact that driver mutations target signaling and molecular *pathways* (Vogelstein and Kinzler, 2004; Vogelstein et al., 2013), groups of interacting proteins and genes performing specific functions in a cell. Mutations in genes belonging to cancer pathways lead to the acquisition of the biological capabilities (e.g., resisting cell death and inducing angiogenesis) or *hallmarks* (Hanahan and Weinberg, 2000, 2011) featured by cancer cells. A cancer pathway may be altered by mutations in any of its genes, leading to a wide spectrum of mutation frequencies for genes in the same cancer pathway, with one or few genes mutated with relatively high frequency and many genes mutated at much smaller frequency, which may not be sufficient for detection by frequency-based methods. In addition, each cancer genome is exposed to different mutational processes characterized by different combinations of mutations or *signatures* (Alexandrov et al., 2013b; Petljak and Alexandrov, 2016), with different cancer types presenting different mixtures of such signatures (Nik-Zainal et al., 2012a, 2016; Alexandrov et al., 2013a, 2015, 2016). Studying and characterizing mutations at the level of pathways is therefore crucial to deal with heterogeneity for the identification of driver mutations and to identify common themes extending the “rulebook” of cancer (McGranahan and Swanton, 2015), with important implications in prognosis and therapy (Swanton, 2016).

**Figure 2:**
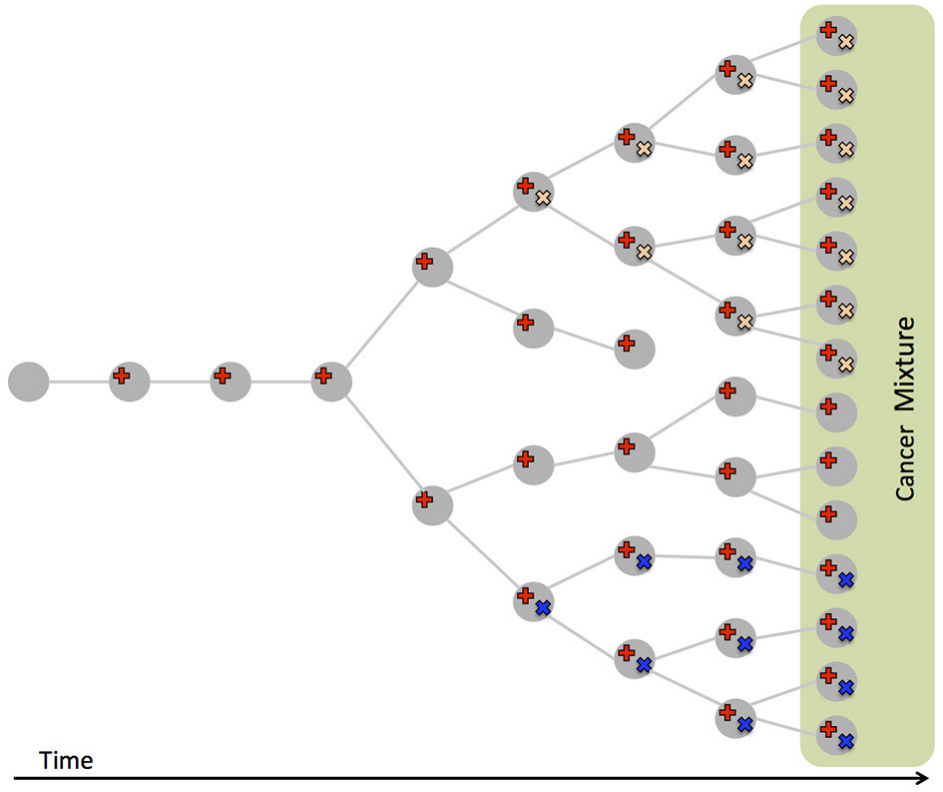
Intra-tumor heterogeneity and its causes. Cancer evolves from a normal cell that accumulates mutations (in red, yellow, and blue), leading to different *clones*, populations of cells of different genotypes, coexisting in the same tumor. Bulk sequencing measures mutations from a sample of the resulting cell mixture, that also comprises normal cells. The fraction of reads supporting a mutation (VAF) is proportional to the number of cells with the mutation in the sample.

In addition to uncover such *inter-tumor heterogeneity*, cancer genome sequencing has also uncovered *intra-tumor heterogeneity* (Figure 2): a tumor is often composed by different populations of cancer cells (Anderson et al., 2011; Gerlinger et al., 2012, 2014; Schuh et al., 2012; Newburger et al., 2013; Bolli et al., 2014; Brastianos et al., 2015; Gundem et al., 2015; Ling et al., 2015; Sottoriva et al., 2015), called *clones*, arising from the evolutionary process (Nowell, 1976) which starting from a normal cell leads, through somatic mutations, to a collection of related but different cancer cells (Greaves and Maley, 2012; Swanton, 2012). While only providing measurements at the level of the entire cell population, deep (e.g., >100-fold) bulk sequencing offers the opportunity to study intra-tumor heterogeneity: the variant allele frequency (VAF), or fraction of reads supporting a variant among all the reads mapped to the same genomic location, of a heterozygous variant in a diploid region is proportional to the fraction of cells with the variant among all cells in the sample. VAFs from a tumor can then be used to identify the various clones present in a tumor. In addition, since the VAFs in a cell are constrained by evolutionary relationships among the clones in a tumor, they can be used to infer the evolutionary trajectory followed by the observed tumor (Ding et al., 2012; Nik-Zainal et al., 2012b; Yates and Campbell, 2012; Burrell et al., 2013). Understanding the clonal composition of a tumor is crucial for prognosis and therapy (Greaves and Maley, 2012; Swanton, 2012), since different clones may present drug resistant mutations (Greaves and Maley, 2012; Swanton, 2012) and the reliable characterization of the evolutionary history of a tumor is needed to predict the development of the disease (Yachida et al., 2010; Lipinski et al., 2016).

A more direct approach to study intra-tumor heterogeneity is single-cell sequencing (Hou et al., 2012; Xu et al., 2012; Wang et al., 2014; Navin, 2015a,b). Single-cell sequencing allows the direct observation of the cooccurrence of mutations within cells from different clones. However, single-cell data is currently noisy, with high false-positive and false-negative rates for mutation calls, and the number of cells that can be assessed is still limited compared to the billions of cells in a tumor.

In this review we describe bioinformatic and computational approaches to characterize cancer heterogeneity from next-generation sequencing data. We consider methods to deal with three aspects of cancer heterogeneity, after alterations such as SVNs and/or CNAs have been identified in a tumor or in multiple tumors (Raphael et al., 2014). First, we consider methods that tackle inter-tumor heterogeneity by characterizing tumor mutations at the pathway level. Second, we describe methods to characterize intra-tumor heterogeneity by using mutations from bulk sequencing or single-cell sequencing. Third, we describe some methods to relate cancer heterogeneity with clinical variables. The computational characterization of cancer heterogeneity is a topic which has spurred a lot of work in recent years and we only cover some of the tools that have been recently proposed. In particular, we only focus on methods assuming that somatic variants have already been called using one of the many methods currently available (e.g., Lawrence et al., 2013), and we refer the reader to other reviews discussing methods for variant calling in cancer (e.g., Raphael et al., 2014). The methods discussed in this review are mostly complementary, describing different characteristics of inter- or intra-tumor heterogeneity, which we believe constitute useful, multi-faceted information for cancer researchers and practitioners.

## 2. Methods for inter-tumor heterogeneity

**Figure 3:**
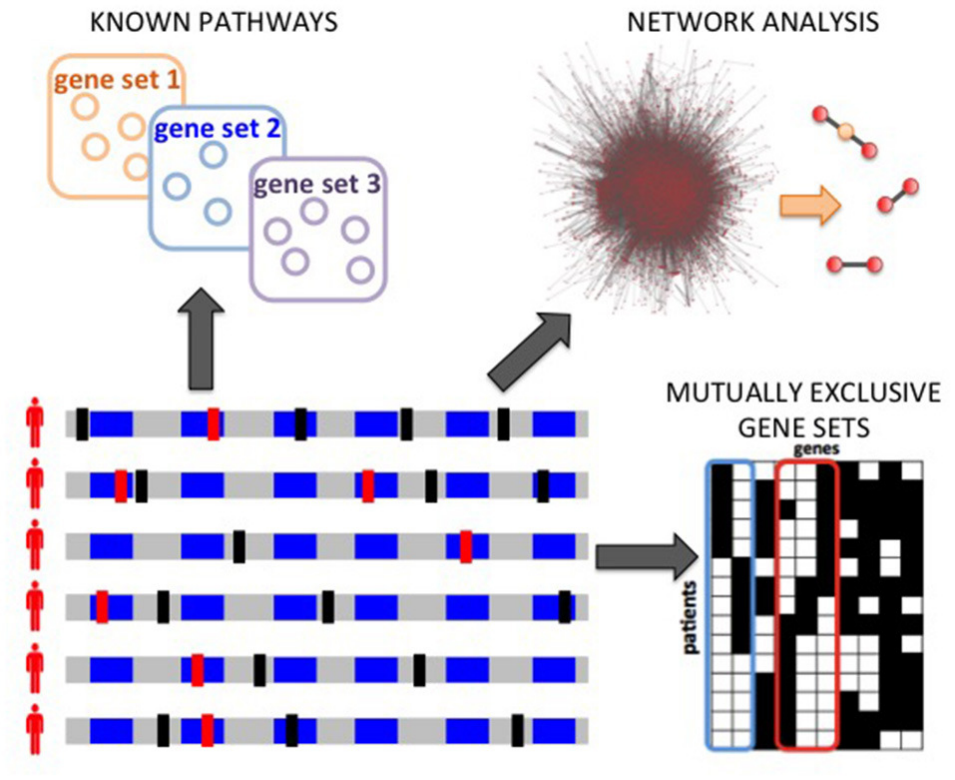
Computational analyses to characterize inter-tumor heterogeneity. Starting from somatic mutations measured in many patients, different types of analyses are possible: annotation and enrichment analysis for known pathways; network analyses to discover significantly mutated subnetworks of a large interaction networks; the *de novo* identification of pathways, based for example on the identification of mutual exclusivity patterns.

Several methods have been designed to characterize inter-tumor heterogeneity by identifying pathways and processes altered in a significant number of patients. These approaches can be categorized into 3 classes (Figure 3): methods based on predefined pathways; methods that extract pathways from a large interaction network of genes or proteins; *de novo* methods that do not use prior information of interactions among genes. Below we review some of the representative methods in each class. In general, the input to each method can be a list of genes mutated in the patients cohort or a score (e.g., frequency of mutation, a score reflecting the significance of the fraction of mutated genes in the cohort; Lawrence et al., 2013, etc.) for each gene in the cohort. As described below, while some of the methods require in input a list of putative driver mutations, identified for example by frequency-based approaches (e.g., Dees et al., 2012; Lawrence et al., 2013), other methods try to leverage the information regarding interactions among genes/proteins to identify novel driver genes which cannot be identified by frequency-based approaches. We highlight here the main methods that produce, in output, pathways, or sets genes summarizing inter-patient heterogeneity, while we do not consider methods which provide instead a ranking of genes (e.g., Vanunu et al., 2010; Shrestha et al., 2014), or which focus on patients stratification (e.g., Hofree et al., 2013), or which combine mutations with other data types (e.g., Vaske et al., 2010; McPherson et al., 2012; Paull et al., 2013). See Creixell et al. (2015) for a more comprehensive review of network approaches to analyze cancer genomes.

### 2.1. Pathway-based approaches

A common way to identify significantly mutated pathways is to use *predefined pathways*, obtained from databases such as KEGG (Kanehisa and Goto, 2000; Kanehisa et al., 2015, 2017) and MSigDB (Subramanian et al., 2005; Liberzon et al., 2015), an then asses whether the set of genes in a predefined pathway is significantly enriched for mutated genes or scores compared to the entire set of genes. The simplest approach is to assess whether a list of mutated genes is enriched for genes in predefined set of genes, for example by using an hypergeometric test on the overlap of the intersection among the list of genes and the gene set. There are several tools [e.g., DAVID (Huang et al., 2009), g:Profiler (Reimand et al., 2016)], some of which originally designed for gene expression data, that can be used for gene lists obtained from mutation data. A common feature of these approaches is that they require the definition of the list of mutated genes, commonly based on thresholds based on frequency or statistical significance of single genes. An alternative is to use Gene Set Enrichment Analysis (GSEA) (Subramanian et al., 2005), a general methodology to assess the association of a ranking of genes with a given gene set. The rank of the genes can be obtained from the various tools mentioned above; for example, Lin et al. (2007) used the Cancer Mutation Prevalence (CaMP) scores (Sjöblom et al., 2006), but other scores can be used. A different approach is taken by PathScan (Wendl et al., 2011), that computes a *p*-value for the enrichment of mutations in a given set separately for each patient, and then combines the *p*-values across all patients. Similarly, the method from Boca et al. (2010) defines, given a gene set, a score for each patient and then combines such scores across all patients.

The methods above are useful to characterize inter-tumor heterogeneity using known sets of genes and pathways, but have some major limitations. First, they require the *a priori* definition of the list of mutated genes, and therefore, while they are useful in organizing a list of mutated genes into pathways, they cannot be used to reliably identify novel driver genes. Second, some of the genes sets from datasets are extremely large (>300 genes). With such large gene sets it may not be possible to identify a small subset associated with the disease. Third, these methods ignore the interactions among genes in a network, considering all genes in a pathway equally, without including the topology of network in their analysis. Fourth, they consider each set of genes as a separate entity, while it is well-known that there is cross-talk among pathways, which interact into larger networks (McCormick, 1999).

### 2.2. Network-based approaches

A different approach to characterize cancer inter-tumor heterogeneity at the pathway level while not restricting to known sets of genes is to use a genome-scale protein-protein interaction network. Several computational methods that combine mutation data and networks to infer gene sets have been designed. A first class of methods (e.g., NetBox; Cerami et al., 2010) looks for significant network modules among a list of genes which is provided as input. Such approaches require to define a score threshold to include genes in the analysis, limiting the possibility of the method to identify novel driver genes. A different approach is to identify significant subnetworks (comprising connected genes) that are significantly mutated in the patients cohort. While allowing to expand from predefined sets of interacting genes to general interacting subnetworks, the identification of significantly mutated subnetwork presents computational and statistical challenges. There is a huge number of subnetworks which need to be screened and which need to be considered into a multiple hypothesis testing framework to identify the significantly mutated ones, therefore naïve approaches (e.g., the enumeration and testing of all subnetworks) cannot be employed and more sophisticated techniques are required.

HotNet (Vandin et al., 2011) and HotNet2 (Leiserson et al., 2015a) address the challenges above by using a diffusion process on a graph to combine gene scores with the network topology while capturing the local structure of the network. A novel statistical test is used by HotNet and HotNet2, allowing the identification of a set of subnetworks while bounding the false discovery rate (FDR). The combination of gene scores and network topology solves the issue of choosing a threshold for the inclusion of genes in the analysis and allows the identification of subnetworks whose significance is due to the mutation scores of the genes *and* the local topology of a subnetwork. In the analysis of >3,000 samples from 12 cancer types from TCGA (Leiserson et al., 2015a), HotNet2 identified 16 significantly mutated subnetworks that comprise well-known cancer pathways as well as subnetworks with less established contributions to cancer, including the cohesin complex.

MEMo (Ciriello et al., 2012) is an algorithm that uses a different approach to identify subnetworks: provided in input with a relative short of list of (frequently mutated) genes from which subnetworks (called modules) are to be found, it identifies groups of genes sharing several neighbors in the interaction network and showing significant mutual exclusivity of mutations in the patients cohort. MEMo therefore identifies modules summarize inter-patient heterogeneity through mutual exclusivity, but it is unlikely to include in its modules genes that are not significantly mutated on their own. MEMCover (Kim et al., 2015) is a different algorithm that combines network information and mutual exclusivity of mutations to identify modules of mutated genes. MEMCover employs a greedy strategy to identify high scoring subnetworks, where a subnetwork score is a combination of the number of patients with at a least a mutated subnetwork member and of the mutual exclusivity of mutations in the subnetwork genes. Babur et al. (2015) present a greedy approach to find gene sets sharing a common down-stream target in the network and showing high mutual exclusivity. They assess mutual exclusivity by comparing each gene in the set with the union of the other genes.

Network-based approaches are useful to characterize inter-tumor heterogeneity without restricting to know sets of genes and pathways, but they suffer from the limitations of currently available network. Such networks have only partial coverage of genes and interactions: some genes have no interactions in current networks, and interactions of different genes may have been assayed to different extents, with genes known to be associated to diseases that are likely to have been more thoroughly assayed for interactions (ascertainment bias). In addition, current networks include interactions that occur among proteins in different tissues or at different phases of the cell cycle. Improved methods are needed to integrate additional information (e.g., co-location of proteins in cells) with the interaction information provided by currently available networks.

### 2.3. *De novo* approaches

Previous approaches are based on knowledge of the interactions among genes/proteins. A different class of methods characterize inter-tumor heterogeneity by finding groups of genes or pathways without restricting to predefined sets or to groups of interacting genes in a large network. The *de novo* extraction of pathways poses enormous computational and statistical challenges, since every subset of genes is a candidate which may need to be considered. However, some methods use combinatorial properties (Yeang et al., 2008) of important mutations in cancer to restrict the set of potential candidates. One such property is *mutual exclusivity*, with sets of genes displaying at most 1 mutation in many patients. Mutual exclusivity of mutations has been observed in various cancer types (Kandoth et al., 2013) and may be due to the relatively low number of driver mutations in each tumor and to the fact that driver mutations target different pathways (Hanahan and Weinberg, 2011; Garraway and Lander, 2013; Vogelstein et al., 2013).

Several methods have been recently designed to identify gene sets with high mutual exclusivity. Since most genes are mutated with low frequency in a cohort of patients, it is easy to find a set of unrelated genes with high mutual exclusivity. For this reason, one needs to assess the statistical significance of the gene set, assessing whether the observed mutual exclusivity is likely to be due to chance alone. RME (Miller et al., 2011) identifies mutually exclusive sets using a score derived from information theory, and starts from pairs of genes to build larger sets. It includes only frequently mutated genes (>10%), limiting its applicability to characterize inter-tumor heterogeneity. Dendrix (Vandin et al., 2012b) defines a gene set score that combines the number of patients with at least a mutation in the set and the mutual exclusivity of mutations in the set, and uses a Markov Chain Monte Carlo (MCMC) approach for identifying mutually exclusive gene sets altered in a large fraction of the patients. Multi-Dendrix (Leiserson et al., 2013) employs the same score as Dendrix and extends it to multiple sets, and uses an integer linear program (ILP) based algorithm to simultaneously find multiple sets of mutually exclusive genes. CoMET (Leiserson et al., 2015b) uses a generalization of Fisher exact test to higher dimensional contingency tables to define a score that better characterizes mutually exclusive gene sets altered in relatively low fraction of the samples, and uses an efficient MCMC approach to identify such sets. WExT (Leiserson et al., 2015b) generalizes the test from CoMET to incorporate individual gene weights (probabilities) for each mutation in each sample, and provides an efficient way to assess the statistical significance of the sets using a saddle-point approximation. Similarly, WeSME (Kim Y.A. et al., 2016) introduces a test which incorporates the mutation rates of patients and genes and uses a fast permutation approach to assess the statistical significance of the sets. TiMEx (Constantinescu et al., 2015) assumes a generative model for mutations and defines a test to assess the null hypothesis that mutual exclusivity of a gene set is due to the interplay between waiting times to alterations and the time at which the tumor is sequenced. The test is used to assess pairs of genes, and larger sets are built from significant pairs and then assessed using the same test. As mentioned above, MEMo and the method from Babur et al. (2015) employ mutual exclusivity to find gene sets, but use an interaction network to limit the candidate gene sets. The method by Raphael and Vandin (2015) and PathTIMEx (Cristea et al., 2016) introduce an additional dimension to the characterization of inter-tumor heterogeneity, by reconstructing the order in which mutually exclusive gene sets are mutated. Kim J.W. et al. (2016) recently developed REVEALER, a method to identify mutually exclusive genes sets associated with functional phenotypes (see Section 4).

While the approaches above allow the *de novo* discovery of cancer gene sets, there are challenges that remain to be solved. For example, larger sample sizes than currently available may be needed to discover low frequency cancer pathways by using mutual exclusivity (Vandin et al., 2012c, 2016). The methods above are in general computationally intensive, mainly due to the large search space that must be explored, and more effective exploration strategies may be needed for larger datasets.

## 3. Methods for intra-tumor heterogeneity

**Figure 4:**
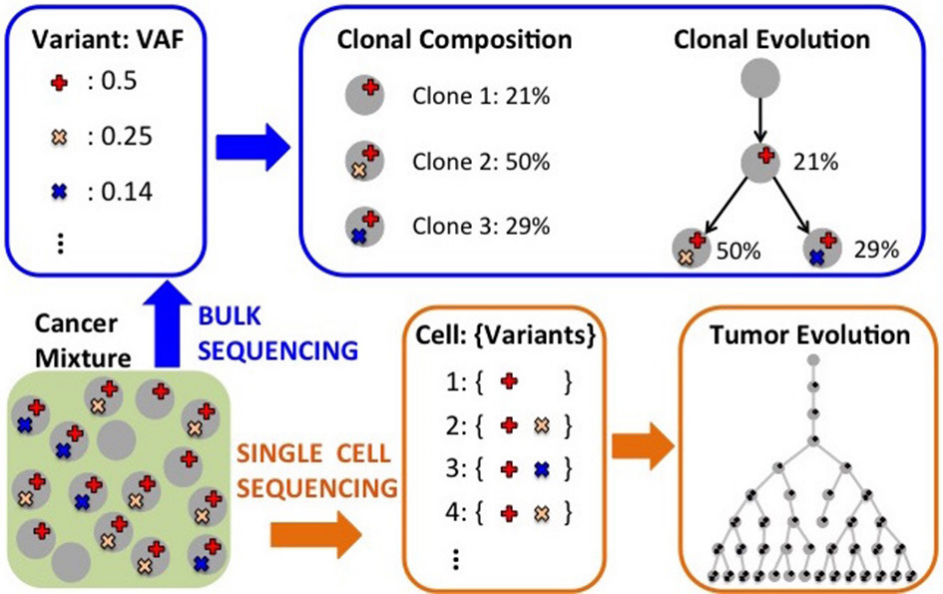
Computational analyses to characterize intra-tumor heterogeneity. Starting from measurements (e.g.,VAFs) obtained from bulk sequencing of one or more tumor samples, one can infer the clonal composition of the sample and also the evolutionary relationships among clones. Single-cell sequencing can be use to infer the evolutionary relationships among the individual cells for which mutations have been assayed.

In recent years, several methods have been proposed to characterize intra-tumor heterogeneity. Such methods can be classified into three classes (Figure 4). First, methods that use mutation data from bulk sequencing to reconstruct the *clonal* composition of a tumor, thus identifying the different *clones*, populations of cells, present in a tumor sample and quantifying the fraction that each clone contributes to the tumor. Second, methods that use mutation data from bulk sequencing to reconstruct the evolutionary relationships among different clones and mutations in the tumor. Third, more recent methods that use mutation data from single cell sequencing to infer the evolution of a tumor at the single cell level. Due to space constraints, below we describe some of the methods in the three classes; we point the reader to the recent reviews by Schwartz and Schäffer (2017) and by Kuipers et al. (2017) for more details on approaches to infer tumor evolution.

### 3.1. Inference of clonal composition from bulk sequencing

Bulk sequencing data provides information regarding the fraction of cells containing a mutation, and, therefore, regarding the fraction of cells defining the clone with the given mutation. In fact, for a heterozygous mutation in a copy neutral region the expected number of reads supporting the mutation (VAF) equals half of the clone frequency in the sample, since the mutation appears in only one of the two copies of the DNA. However, there are many confounders that make the identification of the clones not straightforward. First, the relation above holds only in expectation or for infinite coverage, while with finite coverage the actual VAF can deviate substantially from the corresponding clone frequency. Second, there are experimental biases in sequencing technologies that can change the relation between VAF and clonal frequency. Third, CNAs are quite common in cancer and nullify the relation above, making the inference of clones much more complex. Andor et al. (2016) have recently shown that the number of clones in a tumor is associated with mortality risk, which increases when between 2 and 4 clones are present in a tumor, while it decreases when >4 clones are present. The accurate characterization of the clonal composition of a tumor is therefore extremely important for diagnosis and therapy.

Several methods have been developed to identify the different clones, or cell populations, in a tumor starting from mutation data obtained from bulk sequencing. PyClone (Roth et al., 2014) identifies clones and their abundances by considering VAFs and allele-specific copy number data. It uses a beta-binomial model for VAFs and identifies clusters of mutations and their frequencies in a tumor sample with Bayesian nonparametric clustering which simultaneously infers clusters and the number of clusters. SciClone (Miller et al., 2014) considers VAFs in copy number neutral, loss of heterozygosity (LOH) free regions of the genome, and uses a variational Bayesian mixture model to infer clones and their frequency in the sample. Zare et al. (2014) present an algorithm to infer groups of mutations and their frequency in a tumor using mutation data from multiple sections of a tumor at a given time point. Their method is based a generative binomial model to incorporate information from the multiple sections and employs an expectation-maximization (EM) algorithm to estimate clones and their relative frequencies. BayClone (Sengupta et al., 2015) defines a class of nonparametric models, the categorical Indian buffet process, and uses bayesian inference to obtain posterior probabilities for the number clones, their genotypes, and their proportions, in a tumor sample.

With the coverage (30x–40x) used in many large scale cancer studies, there is a high variance in the number of reads covering a given position in the genome, weakening the relation between VAF and clonal frequency. In contrast, each copy number aberration perturbs many reads, and can provide a more reliable signal for clonal inference for tumors in which clones present different copy number profiles. THetA (Oesper et al., 2013) uses CNAs profiles from whole genome sequencing to characterize clones and their frequencies in a tumor mixture. It defines and optimizes an explicit probabilistic model for the generation of the observed sequencing data from a mixture of normal cells and different clones, and uses a BIC criteria to choose among the many models that may explain the data while balancing the likelihood of the data and the model complexity. THetA2 (Oesper et al., 2014) extends THetA in various directions, including the possibility to consider whole exome sequencing data and the use of B-allele frequencies (which indicates the relative quantity of the one allele compared to the other) to distinguish among several clonal population models consistent with the data. A different approach is taken by TITAN (Ha et al., 2014), which employs a generative factorial hidden Markov model framework to simultaneously infer CNA and LOH segments from read depths and digital allele ratios at heterozygous variant loci in the genome from whole genome sequencing data. CloneHD (Fischer et al., 2014) provides a statistical framework using read depth, B-allele frequencies, and VAFs to infer the clonal population structure of a tumor, allowing the simultaneous analysis of multiple samples from different regions of the same tumor or from longitudinal sequencing of the same tumor.

### 3.2. Inference of clonal evolution from bulk sequencing

While methods to infer clones, their mutations, and their abundance, provide important and clinically relevant insights into intra-tumor heterogeneity, they do not explicitly provide information about the evolutionary relations among mutations and clones in a tumor. In addition to expanding our understanding of how a tumor arises, such information can provide extremely important information for clinical intervention. For example, the order in which mutations arise can influence the prognosis of a patient (Ortmann et al., 2015). Moreover, the characterization of the evolutionary paths followed by tumors is crucial to be able to predict the development of the disease for future patients (Yachida et al., 2010; Lipinski et al., 2016).

The computational reconstruction of the evolutionary relations among clones in a tumor from bulk sequencing data is a challenging task, due to several reasons. First, we do not directly observe clones in a tumor, but bulk sequencing provide aggregate information, in the form of VAFs, from a mixture of clones. Second, a natural model to describe tumor evolution is provided by phylogenetic or evolutionary trees, but there are in general several evolutionary trees consistent with the data from a single tumor sample. In most cases this may be mitigated by sequencing several sections of the same tumor, but reconciling the information from the different sections is a complex problem. Third, VAFs in regions affected by CNAs and LOH can be significantly different from VAFs of other mutations in the same clone, complicating the reliable identification of clones and their relations.

Many methods have been designed to reconstruct the evolutionary history of a tumor from bulk sequencing of one or more sections of the tumor and address the challenges above. TrAp (Strino et al., 2013) is a method designed to infer clones, their abundance, and clones' evolutionary paths using VAFs for SNVs from a single tumor sample. It first groups together mutations with similar frequencies, and then uses an iterative procedure to build evolutionary paths for such groups, starting from simple (height 1) trees. PhyloSub (Jiao et al., 2014) considers VAFs from deep sequencing experiments to infer the evolutionary relationship of clones, and uses a Dirichlet process prior over phylogenetic trees to group SNVs into clones. It employs Bayesian inference, based on MCMC sampling, to infer a distribution over possible evolutionary trees. PhyloWGS (Deshwar et al., 2015) builds on PhyloSub and allows the reconstruction of tumor evolution from SNVs and CNAs obtained from whole genome sequencing data. CITUP (Malikic et al., 2015) proposes a combinatorial model for the problem of inferring clonal evolution from SNVs obtained from multiple tumor samples, and designs an exact algorithm based on a quadratic integer programming to solve the problem, which may require high computational resources when the tumor contains a large number of clones. LICHeE (Popic et al., 2015) is another method to reconstruct clones, abundances, and their evolutionary relationships starting from SNVs measured in multiple samples of a tumor. LICHeE first groups SNVs and identifies clusters of SNVs based on VAFs, and then uses a network to represent VAFs constraints imposed by the evolutionary process. It then identifies an evolutionary model by looking for the spanning tree that best supports the cluster VAF data. BitPhylogeny (Yuan et al., 2015) provides a probabilistic framework that allows the joint inference of the number and composition of clones in a tumor, as well as the most probable tree representing their evolutionary relationship. SPRUCE (El-Kebir et al., 2016) infers evolutionary trees jointly from SNVs and CNAs from multiple tumor samples, with CNAs that are modeled as multi-state alterations, in which alterations can only mutate to a given state at most once in the tree. SPRUCE starts from clusters of SNVs and copy number mixing proportions, and derives a compatibility graph describing the compatibility of state trees for pairs of clusters. The evolutionary trees compatible with the input data are derived by enumerating all spanning trees with appropriate constraints in a labeled multi-graph constructed starting from the compatibility graph. The application of SPRUCE on real data show that many evolutionary trees are compatible with data from multiple samples, cautioning on drawing strong conclusions on any single such tree (Hu and Curtis, 2016). Canopy (Jiang et al., 2016) is a related method to infer evolutionary trees using both CNAs and SNVs from one or more samples, but it starts from raw copy number ratios estimated from CNA segmentation programs. It uses a statistical model and a MCMC algorithm to sample from the space of evolutionary trees, providing a confidence assessment from the posterior distribution. Additional methods to infer clonal evolution are presented in Hajirasouliha et al. (2014), Donmez et al. (2016), Qiao et al. (2014), and El-Kebir et al. (2015).

While each method displays specific features addressing one or more of the challenges above, they are all based, in one form or the other, on the infinite-site assumption: the same site is not mutated twice during the evolutionary history of a tumor. Such assumption may be violated in tumors with high genomic instability, undermining the accuracy of the inferred evolutionary trees. However, without such assumption the inference problem becomes computationally intractable even assuming perfect knowledge of mutations in each clone.

### 3.3. Inference from single cell sequencing

While bulk sequencing provides some information to infer the evolutionary tree describing a tumor history, the best way to elucidate such history is from single-cell data, which provides direct measurements for some of the leaves of a tumor evolutionary tree. Single-cell sequencing technology has been improving in recent years and datasets with SNVs from >40 single-cells are now available (Hou et al., 2012; Xu et al., 2012; Wang et al., 2014). However, mutation calls from single-cell sequencing still suffer from high false positive and false negative rate and missing values, due to various technical reasons (e.g., allele dropout; Kuipers et al., 2017). In addition, while obtaining measurements from hundreds of single-cells is an incredible advance, such cells still represent an extremely small fraction of all cells in a tumor (>10^9^ in advanced tumors). For these reasons, standard phylogenetic approaches cannot be used to infer evolutionary trees from single cell data.

Few methods have been designed to infer the evolutionary relationships among single cells. Youn and Simon (2011) develop a method to infer a *mutation tree*, in which each node corresponds to a mutation and the tree relations describe the relative order among the appearance of mutations in a sample. The mutation tree is reconstructed by using a pairwise test to define the order for pairs of mutations. While the restriction to pairs of genes makes the method efficient, it discards the information among high order relations among mutations. SCITE (Jahn et al., 2016) identifies evolutionary trees from noisy and incomplete mutation data from single-cell sequencing. SCITE uses a statistical model and an MCMC approach to sample trees, error rates, and placement of single cells in the tree. While providing interesting insights, the method is fairly expensive computationally, allowing proper inference only for the limited number of cells available in current datasets. OncoNEM (Ross and Markowetz, 2016) is a related method that uses a nested effects model for the data and employs a heuristic local search algorithm to explore possible tree topologies. While appropriate for current dataset sizes, for much larger dataset such a search algorithm may be too expensive.

## 4. Assessing the association of cancer heterogeneity with clinical variables

A major goal in characterizing inter- and intra-tumor heterogeneity is to understand its impact on prognosis and therapy. In most case, clinical data has been used after the computational characterization of tumor heterogeneity, as a post-processing step testing whether heterogeneity-related features are associated to or predictive for some clinical variable, mostly survival time. For example: survival data or other clinical information are used to evaluate the results of patients stratification methods (Hofree et al., 2013); Andor et al. (2016) computationally assessed the clonal composition of >1,000 samples of various cancer types and then assessed the association between the number of clones in a sample with overall and progression-free survival; Chowdhury et al. (2014, 2015) designed and used a novel algorithm to reconstruct trees describing cancer evolution from single cell copy number data obtained by fluorescence *in situ* hybridization (FISH), and showed that improved prediction accuracy is obtained for classification tasks (e.g., distinguishing primary vs. metastases in the same patient) when features from the cancer evolutionary tree are considered.

**Figure 5:**
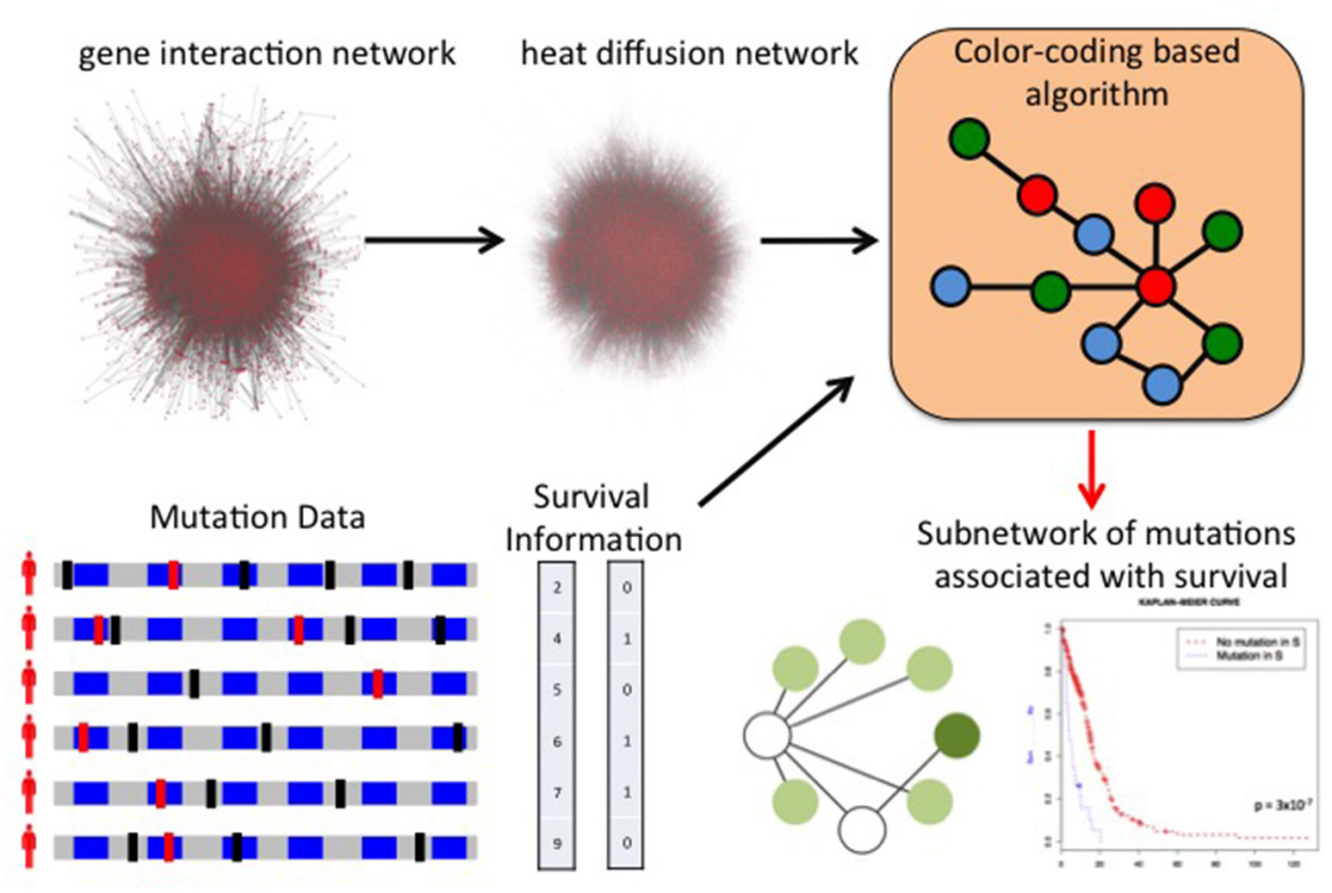
Network of Mutations Associated with Survival (NoMAS). NoMAS combines mutations measured in many patients and the corresponding survival time with a large interaction network to identify subnetworks of genes with significant association to survival. NoMAS is based on an efficient graph color-coding algorithm, and uses a permutation test to correct for multiple hypothesis testing.

The discovery of mutations or mutated groups associated with clinical data starting from genome-wide measurements poses several challenges, due to the peculiar characteristic of genomic data, including the relatively low frequency of individual mutations (Vandin et al., 2015). A standard analysis (Gross et al., 2014) is to first identify driver mutations or pathways and then assess the association of the mutated genes or group of genes with a clinical variable (e.g., survival time). While providing useful information on the clinical relevance of the driver genes and pathway identified by approaches above, such methods may not identify groups of genes with low mutation frequency whose mutations are collectively associated with survival. Few methods have been developed to directly leverage clinical information to identify gene sets associated with clinical data. Vandin et al. (2012a) use gene scores derived from the *p*-values for the association of individual gene mutations with survival as input to HotNet to identify subnetworks associated with survival, but do not provide a method to directly identify gene sets associated with survival. HyperModules (Reimand and Bader, 2013) looks for subnetworks of a large interaction network that are associated with survival using a local search algorithm that builds a subnetwork by starting from one seed vertex and then greedily adds neighbors (at distance at most 2) from the seed. Leung et al. (2014) used it to find subnetworks of a kinase-substrate interaction network with phosphorylation-associated mutations associated with survival. NoMAS (Hansen and Vandin, 2016) is an efficient method based on graph color-coding which identifies subnetworks with mutations associated with survival by looking for subnetworks maximizing the log-rank statistic of subnetworks (Figure 5). NoMAS identifies subnetworks with stronger association with survival compared to greedy procedures, and also reports valid permutational *p*-values. REVEALER (Kim J.W. et al., 2016) is a computational method to identify groups of mutually exclusive genes correlated with a functional phenotype, for example sensitivity to a drug treatment. REVEALER uses a gene set score derived from mutual information and employs a greedy strategy to find genes sets associated with the target functional phenotype.

The methods above provide initial steps to discover gene sets driven by inter-tumor heterogeneity and associated with clinical features, but much more work is required to identify clinically relevant features from tumor heterogeneity.

## 5. Conclusions and future perspective

This review described some of the challenges that arise in studying and characterizing cancer inter- and intra-tumor heterogeneity. We focused on some computational methods which characterize inter-tumor heterogeneity at the level of pathways, infer intra-tumor heterogeneity from bulk or single-cell sequencing, and identify pathways associated with clinical variables. These and other methods are increasingly used to characterize heterogeneity in large sequencing studies and for individual patients. Given its importance for therapeutic decisions, the fast and precise characterization of cancer heterogeneity is likely to remain a key step in precision medicine.

The methods we described have significantly advanced our understanding of cancer heterogeneity and its importance in patient prognosis and treatment, but there still challenges to be addressed. First, while recent studies have shown that intra-tumor heterogeneity has clinical implications (McGranahan and Swanton, 2015, 2017; Andor et al., 2016), it is still unclear which ones among its features are key determinants for therapeutic decisions. The development of more precise computational methods to infer intra-tumor clonal composition and evolution is a necessary step to properly assess the relevance of each aspect for therapy and inform effort for noninvasive monitoring of tumors (e.g., liquid biopsies; Diaz and Bardelli, 2014). Second, the extensive intra-tumor heterogeneity and the stochasticity of some of the processes shaping the evolution of a tumor may limit the ability to accurately predict the future behavior of an individual cancer. Studies (e.g., Jamal-Hanjani et al., 2014) that are collecting molecular and clinical measurements at different time points during treatment for a large number of patients will provide the data necessary to understand the extent of the diversity in the evolutionary paths explored by different tumors, but substantially different computational methods are needed to rigorously and effectively analyze such datasets. Third, current methods for inferring a tumor evolution from single-cell data are computationally intensive, and will not be able to analyze much larger datasets which may soon be available. Fourth, current methods for analyzing bulk sequencing and single-cell sequencing data are orthogonal, but the two technologies provide complementary information about the same tumor. ddClone (Salehi et al., 2017) is a recent method which combines data from the two technologies, but the development of additional methods may be crucial in fully exploiting the power of next-generation sequencing to characterize cancer heterogeneity. Fifth, methods for inter-patient heterogeneity focus mostly on coding variants, while noncoding variants are known to be recurrently mutated in cancer (Weinhold et al., 2014; Melton et al., 2015; Puente et al., 2015), with the mutation in the promoter region of the TERT gene in melanoma (Huang et al., 2013) and other cancer types (Fredriksson et al., 2014; Melton et al., 2015) being a prominent example. Finally, other data types, including RNA sequencing, methylation data, and chromatin modifications need to be considered to understand the genomic heterogeneity of cancer. While there are some methods that integrate some of these data types with mutation data (Vaske et al., 2010; McPherson et al., 2012; Paull et al., 2013), additional work is required to characterize cancer heterogeneity by the full integration of the various data types. All these challenges need to be addressed to reach true precision medicine, and computational methods will continue to play a key role in advancing our understanding of cancer heterogeneity.

## Author contributions

The author confirms being the sole contributor of this work and approved it for publication.

### Conflict of interest statement

The author declares that the research was conducted in the absence of any commercial or financial relationships that could be construed as a potential conflict of interest.

## Footnotes

^1^https://cancergenome.nih.gov

^2^http://icgc.org
